# Predictors of outcome and severity in adult filipino patients with febrile neutropenia

**DOI:** 10.1186/2197-425X-3-S1-A253

**Published:** 2015-10-01

**Authors:** REM Villalobos, MG Yu, RP Berba, MMJ Juan-Bartolome

**Affiliations:** Department of Medicine, University of the Philippines - Philippine General Hospital, Manila, Philippines

## Intr

Febrile neutropenia has a significant mortality rate of 5-21.5%, warranting early recognition and appropriate therapy. Worldwide, there is a lack of studies on prognostic factors that predict poor outcome in patients with febrile neutropenia. It is important to identify these factors to recognize patients who will benefit from early aggressive therapy and closer monitoring.

## Objectives

The study aimed to describe the clinical, laboratory, and microbiologic profile of adult Filipino patients with febrile neutropenia, and to determine specific parameters that are potentially associated with severe outcomes, complications, and mortality.

## Methods

This is a retrospective study of adult febrile neutropenia patients admitted at the Philippine General Hospital from January 2010-October 2014. Patients were described in terms of clinical, laboratory, and microbiologic presentation, and stratified according to the presence or absence of severe outcomes. Prognostic factors were then identified using univariate and multivariate logistic regression analysis.

## Results

A total of 115 febrile episodes in 102 patients were identified. Acute Leukemia (48.7%) was the most common underlying disease in both complicated and non-complicated groups. Most patients (50.43%) had infections of the respiratory tract, and gram negative bacteria were the predominant pathogens. The factors that significantly predicted poor outcome in the univariate analysis were non-treatment/relapse of the underlying disease (OR 2.28; 95% CI, 1.04-4.98; p = 0.040), prolonged fever >7 days (OR 3.24; 95% CI, 1.16-9.01; p = 0.024), non-recovery from neutropenia (OR 2.17; 95% CI, 1.01-4.68; p = 0.048), and severe thrombocytopenia < 50,000/uL (OR 3.45; 95% CI, 1.52-7.84; p = 0.003.) Meanwhile, complete antibiotic therapy significantly predicted a better outcome (OR 0.26; 95% CI 0.12-0.57; p = 0.001.) Using the factors that reached significance in the univariate analysis, subsequent multivariate analysis showed prolonged fever (OR 2.43; 95% CI, 0.77-7.74), positive cultures (OR 2.69; 95% CI, 1.04-6.98), and nadir absolute neutrophil count/ANC < 100 during admission (OR 1.96; 95% CI, 0.75-5.12) as significant predictors of poor outcome. The factors that significantly correlated with better outcome were granulocyte colony-stimulating factor (G-CSF) use (OR 0.31; 95% CI, 0.11-0.85) and complete antibiotic therapy (OR 0.26; 95% CI, 0.10-0.67.)

## Conclusions

Adult febrile neutropenia patients with prolonged fever >7 days prior to admission, positive cultures, and nadir ANC < 100 during admission were at significant risk of developing worse outcomes, whereas G-CSF use and complete antibiotic therapy were significantly associated with better outcomes. These prognostic variables might be useful in identifying patients that need more intensive treatment and closer monitoring.

